# Rapamycin Re-Directs Lysosome Network, Stimulates ER-Remodeling, Involving Membrane CD317 and Affecting Exocytosis, in *Campylobacter Jejuni*-Lysate-Infected U937 Cells

**DOI:** 10.3390/ijms21062207

**Published:** 2020-03-23

**Authors:** Barbara Canonico, Erica Cesarini, Mariele Montanari, Gianna Di Sario, Raffaella Campana, Luca Galluzzi, Federica Sola, Ozan Gundogdu, Francesca Luchetti, Aurora Diotallevi, Wally Baffone, Antonio Giordano, Stefano Papa

**Affiliations:** 1 Department of Biomolecular Sciences, University of Urbino Carlo Bo, 61029 Urbino, Italy; erica.cesarini@uniurb.it (E.C.); mariele.montanari@uniurb.it (M.M.); gianna.disario@uniurb.it (G.D.S.); raffaella.campana@uniurb.it (R.C.); luca.galluzzi@uniurb.it (L.G.); f.sola@campus.uniurb.it (F.S.); francesca.luchetti@uniurb.it (F.L.); aurora.diotallevi@uniurb.it (A.D.); wally.baffone@uniurb.it (W.B.); stefano.papa@uniurb.it (S.P.); 2 Department of Pathogen Molecular Biology, London School of Hygiene & Tropical Medicine, London WC1E 7HT, UK; Ozan.Gundogdu@lshtm.ac.uk; 3 Sbarro Institute for Cancer Research and Molecular Medicine, Center for Biotechnology, Temple University, Philadelphia, PA 19122, USA; giordano@temple.edu; 4 Department of Medical Botechnologies, University of Siena, 53100 Siena, Italy

**Keywords:** *Campylobacter jejuni*, rapamycin, ER-remodeling, lysosome positioning and exocytosis, CD317/tetherin

## Abstract

The Gram-negative *Campylobacter jejuni* is a major cause of foodborne gastroenteritis in humans worldwide. The cytotoxic effects of *Campylobacter* have been mainly ascribed to the actions of the cytolethal distending toxin (CDT): it is mandatory to put in evidence risk factors for sequela development, such as reactive arthritis (ReA) and Guillain–Barré syndrome (GBS). Several researches are directed to managing symptom severity and the possible onset of sequelae. We found for the first time that rapamycin (RM) is able to largely inhibit the action of *C. jejuni* lysate CDT in U937 cells, and to partially avoid the activation of specific sub-lethal effects. In fact, we observed that the ability of this drug to redirect lysosomal compartment, stimulate ER-remodeling (highlighted by ER–lysosome and ER–mitochondria contacts), protect mitochondria network, and downregulate CD317/tetherin, is an important component of membrane microdomains. In particular, lysosomes are involved in the process of the reduction of intoxication, until the final step of lysosome exocytosis. Our results indicate that rapamycin confers protection against *C. jejuni* bacterial lysate insults to myeloid cells.

## 1. Introduction

The lysosome is the terminal component of the endocytic pathway, which possesses a series of biological functions including endocytosis, exocytosis, macropinocytosis, plasma membrane repair, defence against pathogens, cell death, signal transduction, and autophagy [1]. Autophagy has been proposed to be a component of the innate immune response against several types of intracellular microorganisms [2]. In physiological conditions, autophagosomes form and successfully fuse with lysosomes at baseline rates, underling the ability of autophagy to support normal cellular functions. In the presence of an autophagic stimulus, the rate of autophagosome formation, autophagosome–lysosome fusion, and lysosomal degradation increases, resulting in accelerated degradation of autophagic substrates [3]. In fact, autophagy stands out as a key process for the lysosomal degradation of cytosolic entities that is highly interconnected with several other biological functions.

Although in many cases autophagy contributes to cell death, it has a protective and pro-survival function. Autophagy and apoptosis are two fundamental biological mechanisms that may cooperate or be antagonistic, and both are involved in deciding the fate of cells in physiological or pathological conditions.

The functional relationship between apoptosis (‘self-killing’) and autophagy (‘self-eating’) is intricate. Under certain circumstances, autophagy constitutes a stress adaptation that avoids cell death and suppresses apoptosis, whereas in others, it represents an alternative killing mechanism which is activated when apoptosis cannot be executed [4,5]. It is essential to determine which mechanism has to be activated as we try to either protect the cells from cell death, as in neurodegenerative diseases, or induce cell death, as in cancer treatment [6]. Other studies linked autophagy to facilitating the mitochondrial permeability transition: both apoptosis and autophagy could permit this process to cause cell death [7]. Depending on the upstream signals and the different instances, cells activate apoptosis or autophagy, or both, by combining or switching between them in a mutually exclusive manner [8] It is difficult to predict if autophagy is positive or negative: it depends on what gets degraded under different circumstances. Rapamycin, an antibiotic discovered as an anti-fungal agent in the 1970s, is an efficient inhibitor of mammalian target of rapamycin (mTOR) resulting in autophagic induction [9]. In mammals, mTOR is the central player in autophagy signaling. It is a serine/threonine kinase which inhibits autophagy by controlling both translation and transcription of autophagy-related (Atg) proteins, therefore interfering with the formation of autophagosomes. Recent studies showed that mammalian target of rapamycin complex 1 (mTORC1) signaling acts with the ER stress response and contributes to the cell fate decision [10,11,12].

Furthermore, the host cell plasma membrane is a central element in pathogen–host associations with interaction at the cell surface leading to internalization, while intracellular membranes play a critical role in subsequent pathogen trafficking. Ultimately, such pathogen–membrane lipid interactions work coordinately to mediate cytotoxicity [13]. CD317/tetherin (also called tetherin, BST2 or HM1.24) is a lipid raft-associated protein with several protective and deleterious roles. Despite its localization to lipid rafts, CD317/tetherin is internalized via clathrin-dependent endocytosis [14]. Mature CD317/tetherin recycles between the plasma membrane, endosomes, and trans-Golgi network (TGN) [15,16]. Regulation of virus restriction and tumor aggressiveness are the most studied aspects of CD317/tetherin function, where CD317/tetherin is expressed in airway and mucosal epithelia in response to viral infection [17]. Research has focused on the involvement of CD317/BST2 in cancer cells and cancer biology re-evaluation of the overall role of CD317/tetherin in host protection, as it appears that CD317/tetherin has pleiotropic effects in the host. In addition, it has been recently demonstrated [18] that BST2-mediated signaling may be involved in the balance of apoptosis and autophagy: the latter is a process representing a critical mechanism that regulates the fate of infected cells. CD317 might attach exosomes both to the plasma membrane and to each other: it acts to inhibit the spread of certain enveloped viruses, by cross-linking the virions and holding them together at the plasma membrane [19]. Edgar et al. have demonstrated that tetherin might act in a similar manner on exosomal vesicles [20], decreasing CD317’s ability to reduce the number of exosomes associated with the plasma membrane of HeLa cells, with a concomitant increase in exosomes released into the medium.

*Campylobacter jejuni* is the most common causative agent of foodborne infectious illnesses in humans [21]. This Gram-negative bacterium is able to establish commensalism in several animal hosts and promote human diarrheal disease, and *C. jejuni*-associated enterocolitis is typically linked with a local acute inflammatory response that involves intestinal tissue damage [22].

*C. jejuni*, like several other Gram-negative bacteria, produces the cytolethal distending toxin (CDT), which is an important virulence factor that has been characterized in detail [23,24,25,26,27,28,29]. CDT is encoded by the *cdt*ABC operon and has three components: CdtA, CdtB, and CdtC. CdtB is a DNAse I that leads to DNA double-strand breakage in the nucleus, resulting in cell cycle arrest at the G2/M phase, thereby inducing cell distension [30], target-cell death, and/or autophagy. CdtA and CdtC assemble a tripartite complex with CdtB, and act as carriers for delivering CdtB into the cell [31]. *C. jejuni* CDT is released through outer membrane vesicles [32], which fuse with the host plasma membrane via lipid rafts [26], leading to its internalization within the host cells [33]. CdtA and CdtC subunits are only able to bind these cholesterol-rich microdomains on the cytoplasmic membrane, allowing the delivery of the active subunit to cells [34,35,36]. Recently, the carcinogenic potential of *C. jejuni* and the key role of CDT in this process have been demonstrated [37]. CDT is also produced by *S*. *typhi* [38,39], in addition to *Escherichia coli* [23] and other causative agents of chronic infection, such as *Haemophilus ducreyi* [26], *Shigella dysenteriae* [28], *Actinobacillus actinomycetemcomitans* [40], *Helicobacter hepaticus* [27], and other species [41,42]. The damage to the host cells can be mediated either [39,43]: (1) directly by (a) enzymatic attack, (b) DNA damage, or (c) by affecting DNA damage repair mechanisms, or (2) indirectly, by (a) provoking a chronic inflammatory reaction, or (b) producing free radicals. These changes might be associated with carcinogenesis and might stimulate cellular aberrations, modify the immune response, or inhibit normal cell controls. Several studies have indicated that pRb proteins exhibit tumor suppressor activities, and play a central role in cell cycle regulation. In fact, recent data [44] have shown that this protein, although owing to its role in G1/S cell cycle checkpoint, participates in many other cellular functions, including, counterintuitively, the negative regulation of apoptosis cell-cycle activation, and apoptotic inhibition can be directly related to autophagy induction.

We have previously demonstrated that lysates containing CDT from all strains are able to induce endoplasmic reticulum (ER) stress in monocytes, suggesting that ER stress was not associated with CDT, but with other *C. jejuni* virulence factors [45]. In the present study, ER was investigated in U937 cells treated by lysates and with the addition of rapamycin (RM), in association with lysosomes, in the mechanisms of escape from lysate intoxication. U937 cells were adopted secondarily to monocytes [45]. In fact, as they are known to be effective producers of both pro- and anti-inflammatory cytokines, monocytes play a major role in innate immunity and in non-specific host response against both exogenous pathogens—primarily by phagocytosis—and endogenous substances created by tissue damage [46]. Furthermore, other researchers [47,48,49] adopted U937 monocytic cells as a model to study the effects of bacterial infection, particularly for CDT intoxication. Here, we describe the evidence that rapamycin reduces CDT effects by the involvement of membrane CD317/tetherin. Furthermore, RM deeply delays the proliferation of intoxicated U937 cells and rescues them from apoptosis, redirecting the lysosomal compartment and their positioning. Finally RM stimulates ER-remodeling, concomitantly to the impairment of the usual progression of apoptosis and proliferation, with all events induced by the presence of lysates containing CDT.

## 2. Results

To investigate the efficacy of the CDT lysates isolated from the *C. jejuni* wild-type and mutant strains in U937 cells, cytometric and confocal analyses were conducted. In the first part of the work, we report specific results able to highlight CDT activity and to underline cell processes subsequently modified by rapamycin. In the second and wider part of the article, RM mechanistic effects on *Campylobacter jejuni*-lysate-infected U937 cells were evaluated, pointing out the processes and cellular compartments involved.

Although the mTOR inhibitor rapamycin does not exert the complete inhibition of mTORC1 downstream targets [50], we adopted this drug, because it is a macrocyclic triene antibiotic that is produced by fermentation of *Streptomyces hygroscopicus*. It is commercially available and the United States (US) Food and Drug Administration (FDA) approved its employment in the United States. It is used alone or in combination with other drugs in cancer [51], autoimmune diseases [52] and, recently, [53] in bacterial infections.

### 2.1. Morphological Features, Cell Death, Absolute Count, and Evaluation of Cellular Division

**Figure 1:**
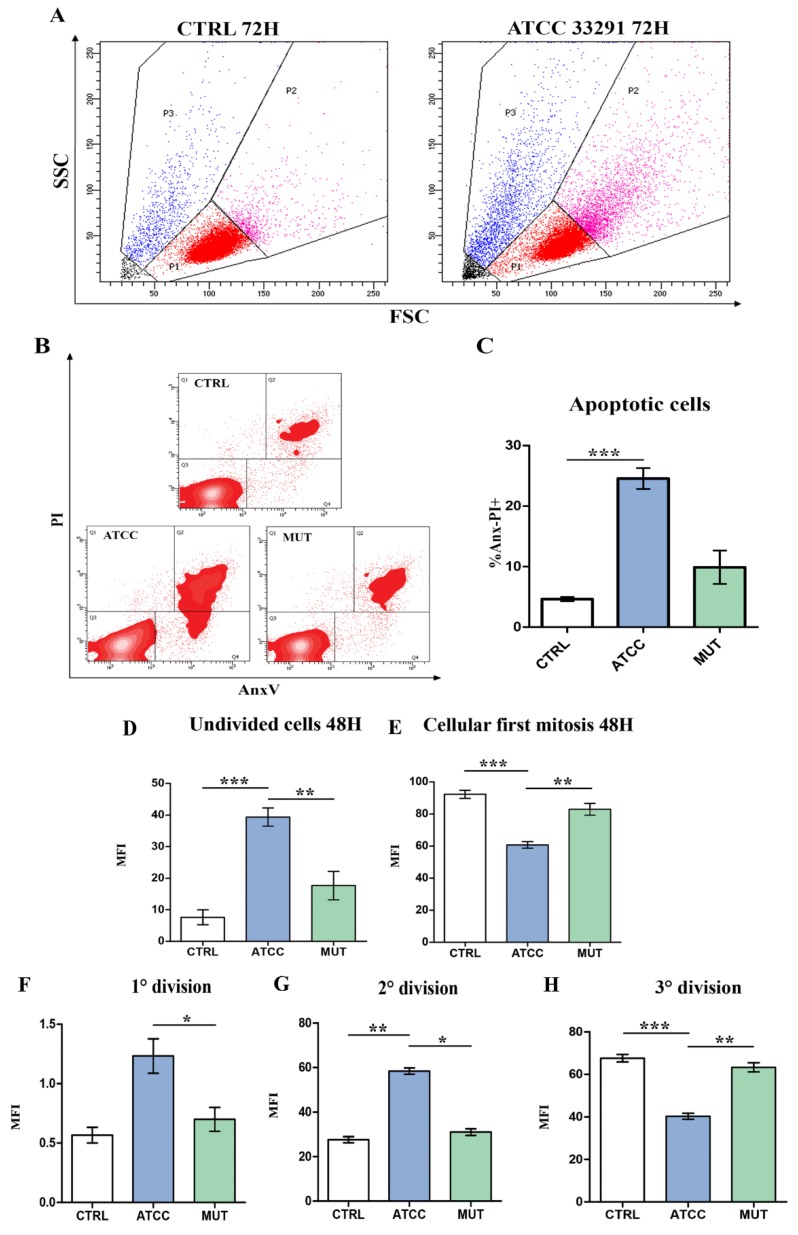
Evaluation of cell death induced by lysates and verification of the efficiency of the toxin, verified only in the ATCC33291-treated cells (**A**) U937 were split into different sub-populations depending on the morphologic parameters: blue gate shows dead cells, pink gate shows distended cells, and red gate shows viable cells. Total cells were the summary of red, pink, and blue gates. Black represents debris which were excluded for the analysis. Dot plots in the picture show control (CTRL) untreated cells and cells treated with the *C. jejuni* ATCC 33291 lysate for 72 h. (**B**) Density plot of propidium iodide (PI) vs. Annexin V (AnxV) of all experimental conditions. Q1 shows PI positive cells, Q4 shows AnxV positive cells, Q2 shows AnxV-PI positive cells, Q3 shows AnxV-PI negative cells and debris. (**C**) Statistical histograms of the percentage of AnxV-PI positive cells calculated after 72 h from lysate administration in total cells. Each value is expressed as a percentage ± SD (results from *n* ≥ 3 independent experiments). Asterisks denote a statistically significant difference (*** = *p* < 0.001) between strains. Statistical histograms related to CFSE dye dilution assay used to determine the number of divisions a given CFSE-labeled cell has undergone. (**D**) Statistical histogram of undivided cells calculated via CFSE staining at 48 h. (**E**) Statistical histogram of dividing cells calculated in cytometry via CFSE staining at 48 h. (**F–H**) Statistical histogram of cells in 1^st^, 2^nd^ and 3^rd^ division, at 72 h. Each value is expressed as a mean ± SD (results from *n* ≥ 3 independent experiments). Asterisks denote a statistically significant difference (* = *p* < 0.05, ** = *p* < 0.01, *** = *p* < 0.001) between strains.

As previously observed in monocytes, morphologic appearance was studied by microscopic and cytometric investigations. Also in U937 cells, *C. jejuni* wild-type lysate caused the typical CDT-dependent cellular distension, compared with untreated control cells and cells treated with the mutant strain lysate. Different sub-populations are visible on the basis of cellular scatter characteristics: we can distinguish normo-sized cells (red events—P1), distended/enlarged cells (pink events—P2), shrunken cells (blue events—P3) (Figure 1A), since one of the main effects of CDT is indeed cell distension/enlargement. Trypan blue viability test and absolute counting beads were applied, revealing that the wild-type strain caused a reduction in cell number in U937 cells (as well as for monocytes and HeLa cells [45,54]). In addition, Annexin V–propidium iodide (AnxV-PI) double staining revealed that AnxV-PI positive cells, which represent the apoptotic cells, were particularly present in U937 cells preincubated with the *C. jejuni* ATCC 33291 lysate (Figure 1B,C).

As shown in (Figure 1D–H), efficiency of CDT in G2-M blocking was investigated by means of carboxyfluoresceinsuccinimidyl ester (CFSE), a fluorescent dye that can measure cell proliferation using flow cytometry. Results at 48 h showed that the *C. jejuni* ATCC 33291 lysate exerted maximally this blocking-effect, showing percentages of undivided cells higher than cells treated with the lysate from the mutant strain. Moreover, via CFSE fluorescence cytometric evaluation, the efficiency of CDT in G2-M blocking was calculated at 72 h (Figure 1F–H)). Bar graphs regarding the first, second, and third division indicated that the typical CDT effect on DNA not only persisted after 72 h from lysate administration, but also became more evident, particularly for the *C. jejuni* ATCC 33291 lysate.

### 2.2. ER Stress Evaluation

**Figure 2:**
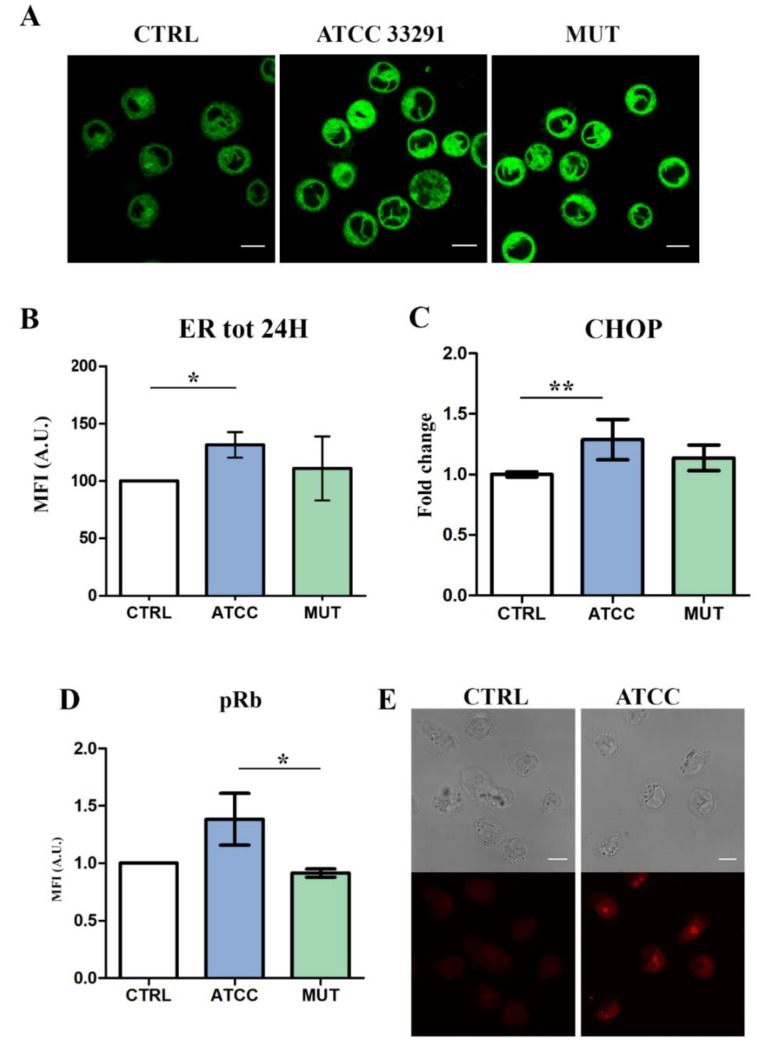
Evaluation of ER involvement by confocal microscopy, flow cytometry, and RT-PCR analyses. (**A**) Single confocal optical sections of ER Tracker MFI of untreated cells and treated U937 at 24 h. Bars: 10 µM. (**B**) Statistical histograms of ER Tracker MFI at 24 h, quantitated by flow cytometry. Each value was converted to arbitrary units (A.U.), setting the control as 100. Each value is expressed as a mean ± SD (results from *n* ≥ 3 independent experiments). (**C**) Evaluation of C/EBP-homologous protein (CHOP) expression in U937 cells after 24 h from lysate administration. Welch’s unpaired *t* test revealed: ** *p* = 0.009 for CTRL vs. ATCC 33291. Evidence of pRb modulation by flow cytometry and confocal microscopy. (**D**) Statistical histograms of pRb intracellular content in U937 cells at 72 h. Each value was converted to arbitrary units (A.U.), setting the control as 1. Each value was expressed as a mean ± SD (results from *n* ≥ 3 independent experiments). The asterisk denotes a statistically significant difference (* = *p* < 0.05) between strains. (**E**) Single confocal optical sections of pRb MFI of untreated cells and U937 cells preincubated with the *C. jejuni* ATCC 33291 lysate for 72 h. Bars: 10 µM.

The ER is a critical stress-responsive organelle, where ER stress leads to the production of nuclear C/EBP-homologous protein (CHOP), which has been implicated as a key mediator of ER stress-mediated cell damage [55]. Unfolded protein response (UPR) is indeed a stress response pathway that promotes the inflammatory response and plays a critical role in a wide range of cellular pathologies [56]. At first, in order to investigate ER stress induced by lysates, U937 cells were stained with ER-Tracker (a live cell stain highly selective for the ER) and analyzed in both cytometry and confocal microscopy. The results reported in Figure 2A–C show that ER stress occurred in U937 cells particularly after 24 h. In addition, having observed significant variations in ER Tracker MFI (Mean Fluorescence Intensity) at 24 h in cells preincubated with the *C. jejuni* ATCC 33291 lysate, CHOP gene expression was quantitated at the same time. As shown in Figure 2C, CHOP expression was significantly induced after 24 h treatment with *C. jejuni* ATCC 33291 lysate.

### 2.3. Prb Detection

Retinoblastoma protein (pRB) has been shown to regulate glucose tolerance, mitogenesis, glutathione synthesis, and the expression of genes involved in central carbon metabolism [57]. Several studies have indicated that pRb proteins exhibit tumor suppressor activities, and play a central role in cell cycle regulation [58]. In fact, recent data [44] have shown that this protein, although owing to its role in the G1/S cell cycle checkpoint, participates in many other cellular functions, including, counterintuitively, negative regulation of apoptosis. 

Changes in pRb (Figure 2D-E) highlight a significant increase between ATCC and mutant-treated cells. Our data show an overexpression of pRb in cells after an incubation of 72 h with the wild-type lysate, compared with the control and the mutant. Confocal analyses focused on the nuclear localization of pRb, well evident after the ATCC 33291 lysate administration. We can consider a pRB-dependent G_2_ arrest (induced by toxins), accordingly to data from the literature [59]. This process has features of noncanonical cell cycle blocker function, normally explicited in the G1/S cell cycle checkpoint, able to reduce proliferation and apoptosis in *C. jejuni* infected cells [45]. 

### 2.4. Rapamycin Inhibition of MTORC1 Signalling Reduces CDT-Induced Distension, Cell Death and Proliferation

**Figure 3:**
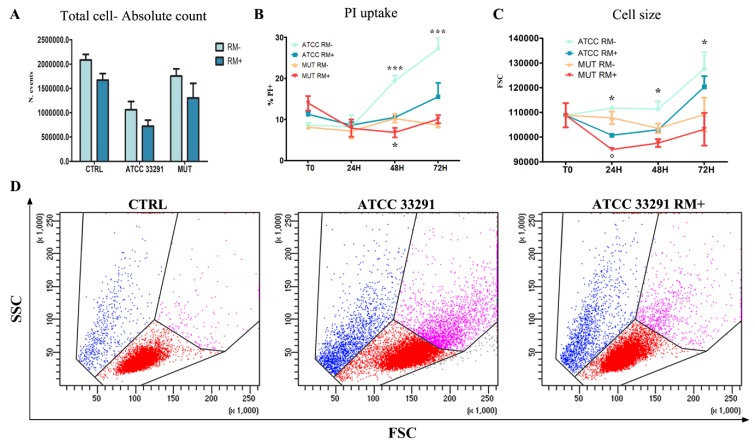
Evaluation of cell count, cell death, and cell size induced by lysates and rapamycin (RM) administration (**A**) Absolute count calculated after 72 h from lysate administration in total cells. Each value is expressed as an absolute number ± SD/mL (results from *n* ≥ 3 independent experiments). (**B**) Trends of percentage of AnxV-PI positive cells calculated from T0 to 72 h from lysate administration, with (RM+) or without RM (RM-). Each value is expressed as a percentage ± SD (results from *n* ≥ 3 independent experiments). Asterisks denote a statistically significant difference (***= *p* < 0.001) between strains. (**C**) Trends of forward light scatter (FSC) values for each treatment during the time course from the starting time point (T0) to 72 h. Each value is expressed as a mean ± SD (results from *n* ≥ 3 independent experiments). Asterisks denote a statistically significant difference (* = *p* < 0.05) between strains. (**D**) Dot plots of scatter characteristics (FSC vs. side scatter (SSC)) of U937 control cells (CTRL) and *C. jejuni* ATCC 33291 U937 treated cells with (ATCC 33291 RM+) or without (ATCC 33291) rapamycin at 72 h. Cellular distension (events in blue) is well appreciable for *C. jejuni* ATCC 33291 U937 treated cells; rapamycin, on the other hand, reduces the amount of distended cells. The gates are drawn around roughly the same areas of Figure 1.

**Figure 4:**
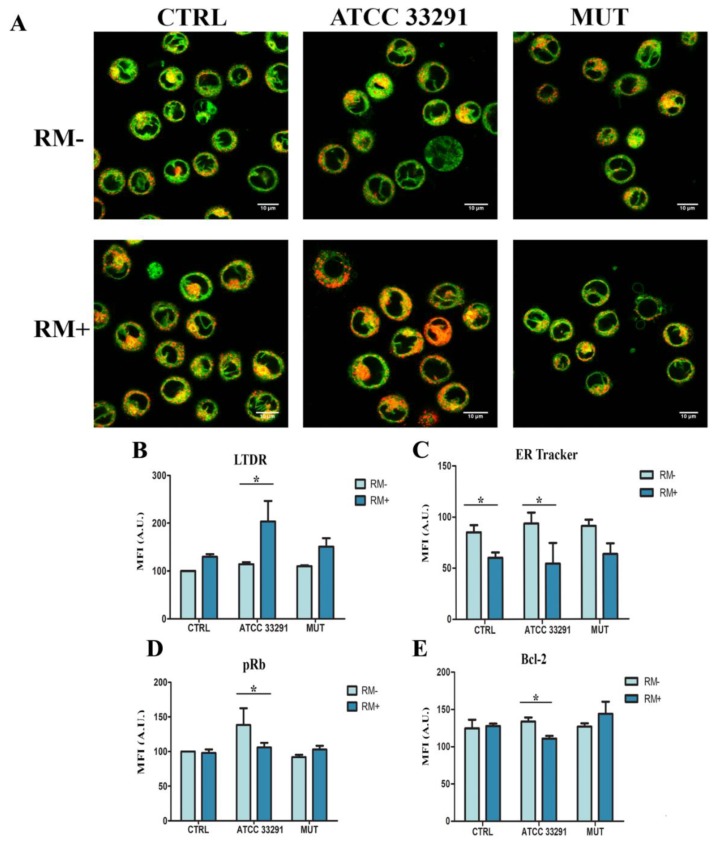
Concomitant evaluation of ER tracker and LysoTracker Deep Red (LTDR) fluorescence by qualitative analysis (confo) (**A**) Single confocal optical sections of ER Tracker (in green) and LTDR (in red) of untreated U937 cells (CTRL) and U937 cells preincubated with lysates after 72 h of treatment with and without RM. Where present, co-localization is shown in yellow-orange. Lower pictures show U937 cells pre-treated with RM for 2 h before lysate administration. Bars: 10 µM. Contemporaneous evaluation of ER tracker and LTDR fluorescence by quantitative analysis (flow cytometry). (**B,C**) For investigated parameters, ER (by ER Tracker staining) and lysosomes (by LTDR staining), a comparison between RM- and RM+ values was carried out for each experimental condition at 72 h. The pRb and bcl-2 expression modulated by lysates (light blue) and lysates + RM (dark blue). (**D**) Statistical histograms of MFI of pRb were calculated after 72 h from lysate administration in distended cells with and without RM. Each value was converted to arbitrary units (A.U.), setting the control as 100. (**E**) Statistical histograms of MFI of bcl-2 was calculated after 72 h from lysate administration in distended cells with and without RM. Each value is expressed as a mean ± SD (results from *n* ≥ 3 independent experiments). Asterisks denote a statistically significant difference (* = *p* < 0.05) between strains.

RM administration can reduce or eliminate important, CDT-induced effects, such as cell size enlargement, degree of apoptosis, and cell cycle perturbation (Figure 3). In particular, this last effect is due to the deep impact of RM in cell growth: It is known [60] that growth is decreased, and also our results on cell count underlined this evidence (Figure 3A), accordingly to pRb data (Figure 4D) Furthermore, RM administration leads to a decrease in apoptotic events, as stated by PI uptake results (Figure 3B). Intriguingly, since mTOR regulation of cell growth and cell size is complex, involving tight regulation of both anabolic and catabolic processes, RM appears to limit the cell enlargement induced by CDT (Figure 3C,D). To exclude that the prevalence of catabolic over anabolic pathways represents the main cause of cell size reduction, we performed the same analytic approach on RM+ and RM- control cells (S1-I): at 24 h and 48 h, RM+ cells demonstrated a slight and not relevant cell size decrease, if compared with RM- control cells. Of note, at the 72-h time point, RM-treated control cells highlighted a moderate increase in cellular dimension (Figure S1-I), whereas in ATCC 33291-treated cells, RM still continued to limit CDT-induced cell distension. (Figure 3C,D). The trend of cell distension/enlargement, evaluated on the basis of the forward light scatter (FSC) cytometric parameter, was also checked by measuring cell size in several single confocal optical sections (Figure S1-II, III). The above-mentioned effects are evident in ATCC 33291 lysate-treated cells, showing that RM specifically antagonizes the efficient CDT produced by this strain.

### 2.5. Rapamycin Stimulates ER-Remodeling, Increases Lysosome Number, Modifies Their Distribution, Decreasing Prb and Bcl-2 Content

Once established the main parameters and cellular pathways in which we expected to highlight a marked role of RM, we evaluated the activation of autophagy by mTOR inhibition, investigating the effects of RM in U937 cells, uninfected or infected with the respective lysates. As shown in Figure 4, significant differences were reported between uninfected and infected cells (by lysates) and untreated and treated cells (by RM). ER stress was significantly demonstrated for the wild-type strain, although mutant treated cells also showed a certain increase of ER tracker MFI and CHOP increase (Figure 2A–C). The additional enlargement that we observed for ER tubules and sheets, in ATCC 33291 lysate-treated cells, may be due to the CDT-dependant rearrangement of the cell size [61] (Figure 4A). Although ER stress and autophagy can function independently, mounting evidence indicates that autophagy is interrelated to the ER at many levels. Cytometric data (Figure 4B) demonstrated that RM, in ATCC 33291 treated cells, significantly and deeply increase lysosomal compartment (traced by Lysotracker Deep Red-LTDR, a fluorescent dye for labelling and tracking acidic organelles in live cells): this event is certainly due to the combination of CDT plus RM, as the inhibitor of mTOR does not increase as much as lysosomes, neither in the control or in the mutant treated cells. Contemporary RM administration decreases ER-tracker fluorescence (Figure 4C), particularly and significantly in ATCC33291-treated cells.

This behavior is accompanied by a decrease in both pRb and bcl2 (Figure 4D,E). In fact, RM pretreatment reduces pRb intracellular content in ATCC 33291 lysate-treated cells: this finding is consistent with the limitation of some effects exploited by CDTs, i.e., cell cycle blocking, enlargement of cell size and autophagosome/autophagolysosome induction. In fact, Rb protein is upregulated by wild-type lysate and subsequently reduced by RM pretreatment. A decrease of the anti-apoptotic protein, Bcl-2, is usually associated with amplified apoptosis; however, as demonstrated in the previous paragraph, RM reduces PI+ events, induced by CDT. Coupling these findings, we identified that in U937 cells, the induction of cell death by CDT is, at least in part, bcl-2-independant and that RM leaves the cells still prone to apoptotic triggers.

**Figure 5:**
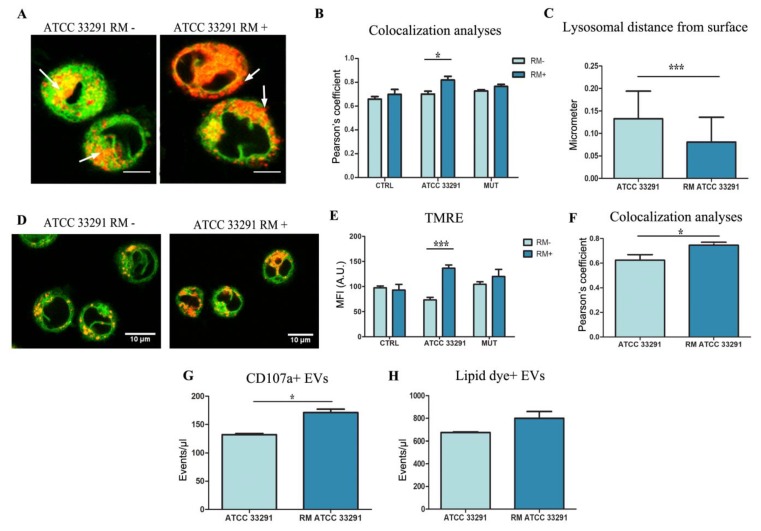
Confocal analyses: ER morhology and extension and its relationship with lysosomes. (**A**) Single confocal optical sections of ER Tracker (in green) and LTDR (in red) of ATCC33291 lysate treated U937 cells and U937 cells preincubated with lysate and treated by RM, after 72 h. Co-localization is shown in yellow-orange and white arrows indicate the positioning of lysosomes, from a perinuclear area (ATCC 33291 RM-) to a peripheral distribution (ATCC 33291 RM+). Bars: 10 µM. In (**B**), Pearson’s coefficient, able to quantitate co-localization, specifically induced by RM in ATCC 33291 lysate treated cells. Asterisks denote a statistically significant difference (* = p < 0.05) between strains. (**C**) Lysosome distance from surface calculated by ImageJ. Calculation performed on 30 cells for each experiment (*n* = 3). Only lysosomes with a puncta and definite morphology were counted, from a minimum of 10/cell to a maximum of 30/cell. Mitochondria membrane potential and ER–mitochondria interactions. Asterisks denote a statistically significant difference (*** = p < 0.001) between strains. (**D**) Single confocal optical sections of ER Tracker (in green) and TMRE (in red) of ATCC33291 lysate treated U937 cells and U937 cells preincubated with lysate and treated by RM, after 72 h. Red fluorescence from mitochondria and their puncta are clearly increased in RM+ cells. Bars: 10 µM. (**E**) TMRE MFI values of RM- and RM+ lysate-treated U937 cells. Asterisks denote a statistically significant difference (*** = p < 0.001) between strains. (**F**) Pearson’s coefficient, able to quantitate TMRE/ER-tracker co-localization, specifically induced by RM in ATCC 33291 lysate treated cells. Total extracellular vesicles (EVs) and EVs from lysosome exocytosis. Asterisks denote a statistically significant difference (* = p < 0.05) between strains. (**G**) Flow cytometric quantitation of CD107a + EVs with (RM ATCC 33291) or without (ATCC 33291). Asterisks denote a statistically significant difference (* = p < 0.05) between strains. (**H**) Flow cytometric quantitation of lipid dye + EVs with (RM ATCC 33291) or without (ATCC 33291).

Confocal analyses were applied not only to confirm the flow cytometric quantification of ER and lysosomal network, but also to underline a possible co-localization of the two compartments, suggested by the flow cytometric data. ER-remodeling behavior is focused by the orange merged-area and quantitated by Pearson’s coefficient. (Figure 5A,B). RM induces a concomitant increase of LTDR MFI and the decrease of ER-tracker MFI was quantitatively calculated by FCM coupled to Pearson’s coefficient evaluated by CM (Figure 5B). Furthermore, by means of TMRE (a potentiometric probe that can be used for direct measurement of the mitochondria membrane potential *∆Ψm*) [45], we evaluated the RM preservation of *∆Ψm* (Figure 5D,E) and concomitant increase in TMRE–ER-tracker co-localization (Figure 5D,F). This increase in ER–mitochondria proximity [62] and in TMRE fluorescence suggests that RM can modulate both mitochondrial bioenergetics and ER–mitochondria contacts. We want to highlight how the quantitative cytometric data help to interpret the co-localization frameworks: in fact, the increase of co-localization for lysosomes and ER happens in parallel to a significant and relevant decrease of ER-tracker fluorescence (Figure 4C) (attesting a decrease in ER *cisternae* and tubules), and a strong increase in lysosome–LTDR fluorescence (for ATCC 33291 lysate-treated cells only).

In Figure 5A, we can observe a diverse positioning of lysosomes, moving from a perinuclear area to a peripheral distribution (arrows). This is an intriguing effect because lysosomal positioning influences mTOR activity, autophagosome biogenesis and autophagosome–lysosome fusion [63]. The availability of lysosomes at the perinuclear region could control the rate of autophagosomal degradation. The observed peripheral lysosome distribution (Figure 5C), induced by RM, is optimal for budding and release via lysosomal exocytosis, as previously demonstrated [45]. Autophagic flux and exosome release are two connected processes that cause loss of cellular membrane and protein content. Figure 5G,H highlights extracellular vesicle count in ATCC33291 lysate-treated cells and RM-treated counterparts. We can observe a moderate and non-significant increase (15%) in lipid dye positive vesicles of RM ATCC samples in respect of ATCC samples, whereas this increase becomes more evident (25%) and significant for CD107a^+^ vesicles, in agreement with the peripheral dispersion of lysosomes. These data underline that RM interferes specifically in lysosomal exocytosis and not mainly on multivesicular body (MVB) formation, budding, and release.

### 2.6. Evaluation of Membrane Microdomains 

**Figure 6:**
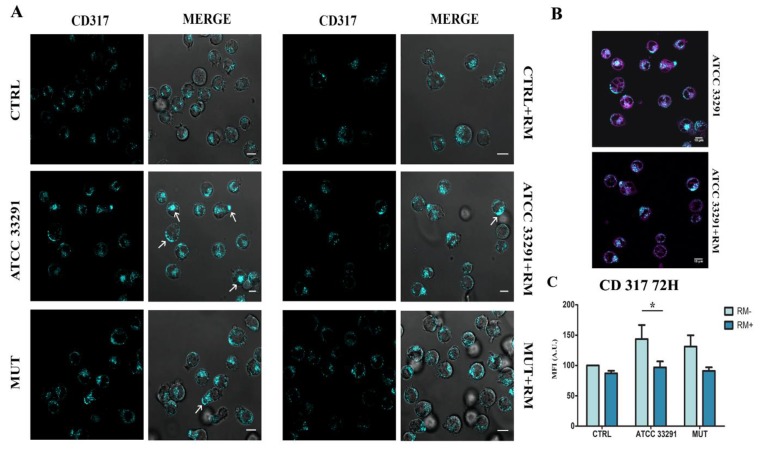
Expression of CD317/tetherin: localization and quantitation by confocal and cytometric analyses, respectively. (**A**) Single confocal optical sections of CD317 of untreated U937 cells (CTRL) and U937 cells after 72 h of treatment by lysates, with and without RM. Bars: 10 µM. (**B**) A comparison between RM- and RM+ ATCC33291 lysate (CDT active)-treated cells: magenta stain represents ER-tracker fluorescence, added to better visualize CD317 distribution and to resemble the ER decrease induced by RM. Bars: 10 µM (**C**) Statistical histograms of CD317 MFI were calculated after 72 h from lysate administration in cells with and without RM. Each value was converted to arbitrary units (A.U.), setting the control as 100. Each value is expressed as a mean ± SD (results from *n* ≥ 3 independent experiments). Asterisks denote a statistically significant difference (* = p < 0.05) between strains.

CD317/tetherin is an organizer of membrane microdomains [64]. CD317/tetherin is an antigen localized within lipid rafts on the cell surface, in the TGN, and/or within recycling endosomes [17]. The different positioning of lysosomes and the extracellular vesicle release, together with its involvement during infections, prompted us to investigate the expression and the distribution of CD317 in our experimental model. As showed in Figure 6C,D, in infected U937, we found an important increase in CD317 expression after 48 h of treatment, whereas RM significantly prevented this effect. Confocal analyses confirm the cytometric data highlighting also a redistribution of CD317 on the cell surface. We observed the formation of a large number of CD317 clusters, in particular in ATCC 33291 treated samples (Figure 6C), whereas in the control condition, this surface antigen appeared to spread on the cell membrane. A similar phenomenon was also observed by other researchers that demonstrated that HIV-1 viral proteins bring to the reorganization of plasma membrane microdomains inducing the coalescence of lipid rafts and tetraspanin-enriched microdomains [65]. As evident in Figure 6C, RM pre-treatment avoided CD317 clusterization and coalescence, appearing similar to the control condition, also in the presence of lysates. It is clear that RM, by modifying the levels and distribution of CD317, plays a role in the organization of lipids in the plasma membrane and in the distribution of proteins that are confined to lipid rafts [66].

## 3. Discussion

In this paper, we studied the effects of RM in *C. jejuni* CDT containing lysate-treated U937 cells. As it was already demonstrated [45,54], *C. jejuni* lysates were able to induce morphologic changes, cell cycle arrest, therefore a reduction in cell number, and apoptosis in different cell models. Here, we demonstrate, for the first time, the powerful action of RM on *C. jejuni* lysate-treated U937 cells, particularly on the mechanisms of CDT activity.

Adopting U937 as a cellular model [47,48,49], we firstly tested CDT activity on U937 cells, examining apoptosis, cell cycle blocking, ER stress, and pRb expression. Indeed, lysosome network involvement and ER-remodeling induction were found as two subcellular processes triggered by RM in saving cells from the action of CDT and, more generally, from the effects of lysates.

At the center of the regulation of mTORC1 signaling is the lysosome, and lysosome distribution influences mTORC1 activity. Intriguingly, the bulk of lysosomes, early and late endosomes, locates quiescently in the perinuclear region of the cell [67,68,69,70,71,72], poised toward the cell’s periphery, as it is shown. Our data display a moderate rise in the lysosome network, primed by lysates, and significantly peaked by the addition of RM. This behavior is particularly evident in cells preincubated with the *C. jejuni* ATCC 332921 lysate, with an active CDT. These findings demonstrate that RM specifically counteracts CDT activity. Contemporarily, after 72 h of treatment, U937 cells respond to stimuli by activating the expansion of the acidic compartment, increasing ER–lysosome contacts and decreasing the ER volume: a scenario strongly suggesting ER-phagy. Late endosomes and lysosomes also make extensive contact with the ER, and numerous studies implicate ER-endolysosomal cross-talk in organelle reshaping and migration [73]. The ER-remodeling and ER-phagy events are selectively activated and they possess the potential to rebalance the ER network and, in our data, might also impact the function of other organelles. It is known that, under basal conditions, ER-phagy occurs constantly at a low level to maintain ER homeostasis [74] and also plays a prominent role in the innate defence against viral and bacterial infections [75,76]. In fact, after the lysate administration, we observed ER stress and ER expansion in cells, and RM, clearly triggering a lysosome increase, strengthening of acidic vacuoles, and ER-remodeling (with features of ER-phagy), represents a powerful treatment to minimize the pathogen attack [77], and it is able to re-establish the pre-stress ER volume, content, and activity [78].

The ER is the only intracellular organelle covering every corner of the cytosol, it has been shown to participate in various functional contacts with other membranous compartments, mediating exchange of metabolites and controlling transport and fusion processes [68,79].

Our findings demonstrate that ER-remodeling (with a key role in the maintenance of ER homeostasis) participates in the cell survival and functional recovery induced by RM. Bravo-Sagua and coworkers [63] showed that ER stress, during its early stage, increases the ER–mitochondria contacts, thus leading to an adaptive increase in mitochondrial energetics. This appears to be a generic response to acute stress, as we similarly observed such changes upon inhibition of the nutrient-sensing kinase mammalian target of rapamycin complex 1 (mTORC1) [80]. In fact, RM enables lysate treated cells to maintain a high mitochondrial membrane potential, accordingly to the decrease of the apoptotic process. Furthermore, RM seems to increase the ER–mitochondria proximity and higher mitochondrial membrane potential, as it appears from FCM and CM analyses of TMRE/ER-tracker labelled samples. 

We detected an increase in the pRb intracellular content, significantly induced by the wild-type strain and partly reverted by RM administration. We found for the first time that this drug is able to partially avoid or delay specific sub-lethal effects, most of these linked to CDT action, able to infer in U937 cells autophagic and ER stress. The pRb decrease (as well as bcl-2 drop) characterizes a cellular status prone to apoptosis. Such effect, together with the downregulation of CD317/tetherin, picked by lysates in the cell surface, represents part of the mechanistic aspects of the results shown in Figure 3. 

pRb has been reported to cause growth arrest and inhibit apoptosis [81]. Upregulation of pRb by the *C. jejuni* wild-type strain might represent a helpful expedient that this bacterium might activate to persist in host cells and to contrast with the programmed cell death. Noteworthy RM addition decreases nuclear pRb content. Interestingly, the recent literature on pRb indicates that loss of pRb function enhances the efficacy of several types of anticancer agents, including tamoxifen, microtubule-interfering agents, and DNA-damaging agents [82]. It has been reported that pRb and lysosomes are interconnected during senescence [83]. The lysosome also extensively communicates with other cellular structures by exchanging content and information and by establishing membrane contact sites. It is now clear that lysosome positioning is a dynamically regulated process and a crucial determinant of lysosomal function. Our results show that RM strongly induces a redirection of lysosomes, from a juxta-nuclear position towards the periphery. Lysosomal positioning is intimately associated with mTORC1 activity and nutrient levels. For example, mTORC1 activation under nutrient-rich conditions causes scattering of lysosomes to the periphery [84,85]. Furthermore, we showed that the RM-induced lysosome redistribution is in connection with vesicle release. The directionality of vesicle transport is regulated also by ER-lysosome contact sites. Currently, contact sites between the ER and endolysosomes are emerging as potent regulatory hubs for vesicle transport [86,87], fusion [88], and fission [89] events. The process of vesicle release in our research is limited to CD107a^+^ extracellular vesicles (EVs), resembling lysosomal exocytosis and clearly increased by RM.

CD317/tetherin (i.e., BST2 or HM1.24 antigen) is an interferon inducible membrane protein present in regions of the lipid bilayer enriched in sphingolipids and cholesterol (often termed lipid rafts). It has been implicated in an eclectic mix of cellular processes including, most notably, the retention of fully formed viral particles at the surface of cells infected with HIV and other enveloped viruses [90]. Edgar and coworkers [20] demonstrated that reduction of tetherin strongly reduces the number of exosomes associated with the plasma membrane with a concomitant increase in exosomes released into the medium. In agreement with these findings, we found an increase of extracellular vesicles in the medium of RM+ ATCC 33291-treated cells, together with a decreased amount of CD317 in the plasma membrane.

In fact, in our model, rapamycin pre-treatment avoids CD317 clusterization and coalescence, previouly induced by lysates, particularly by CDT. RM not only decreases CD317 expression but impacts on its distribution: this molecule displays antiviral (and antibacterial) functions and it is involved in disease manifestation [91]. Therefore, modulating CD317 expression and/or activity has the potential to influence the course of disease.

In summary, *C. jejuni* cytolethal distending toxin impairs apoptosis and lysosomal routes with evidence of ER stress; RM modifies this cellular response to CDT exposition, increasing and redirecting lysosomes, in order to unlock the later stages of lysosomal exocytosis, increasing the release of extracellular vesicles, and the clear reduction and re-assembly of CD317/tetherin.

This research, although with the known limitations of the cellular “in vitro” model, opens up the possibility to use RM as a drug to minimize the risk of the onset of sequelae such as the Guillain–Barré syndrome, in case of massive and important infections by *C. jejuni*. Further aspects are under investigation, to deeply monitor the pathways identified here in the RM control of U937 *C jejuni* lysate intoxication.

A deeper understanding of the modulations and interconnection between these physiological processes, especially under pathological conditions, will be of great importance and may shed light on developing new strategies in treating *C. jejuni* infections.

## 4. Materials and Methods

### 4.1. Cell Culture

U937 (human myelomonocytic cell line) cells (Sigma-Aldrich, St Louis, MO, USA) were grown in RPMI 1640 supplemented with 10% heat-inactivated fetal bovine serum (FBS), 2 mM glutamine, and 1% antibiotics, and was maintained at 37 °C in humidified air with 5% CO_2_.

### 4.2. Growth Conditions of Bacterial Strains and Cell Lysate Preparation

*C. jejuni* ATCC 33291 and *C. jejuni* 11168H *cdtA* mutants were grown at 37b °C in a microaerobic chamber (Don Whitley Scientific, Shipley, United Kingdom) containing 85% N2, 10% CO2, and 5% O2, either on blood agar (BA) plates containing Columbia agar base (Oxoid, Basingstoke, United Kingdom) supplemented with 7% (*v/v*) horse blood (TCS Microbiology, United Kingdom) and *Campylobacter* Selective Supplement (Oxoid), or in Brucella broth (Oxoid) with shaking at 75 rpm. *C. jejuni* strains were grown on BA plates for 24 h prior to use in all assays, unless otherwise stated.

*C. jejuni* strains were grown in 50 mL Brucella broth (Oxoid) at 37 °C in a shaking incubator under microaerophilic condition for 48 h. The bacterial suspensions were centrifuged at 4000 rpm for 10 min and the pellets were resuspended in 20 mL of Dulbecco’s modified eagle medium (D-MEM) (Sigma-Aldrich, St Louis, MO, USA). Then, bacterial suspensions were adjusted spectrophotometrically to approximately 10^8^ bacteria/mL and lysed by sonication (2 × 30 s bursts with 30 s intervals between each burst) by using a sonicator (Sonifier 450, Branson, Danbury, CT, USA). Cell debris and un-lysed bacterial cells were then removed by centrifugation at 4000 rpm for 10 min. Aliquots of each lysate were sterilized by a 0.22-μm membrane filter (Millipore, Milano, Italy) and stored at −20 °C before use [68].

### 4.3. Pre-Treatment of U937 Cells with Rapamycin

RM is an efficient pharmacological inhibitor of mTOR (mammalian target of rapamycin), which is able to induce autophagy in mammal cells [9]; mTOR is a complex composed of two entities, mTORC1 (mTOR complex 1) and mTORC2 (mTOR complex 2); mTORC1 is a downstream target of PI3K (phosphoinositide 3-kinase) and is sensitive to the RM [92]; mTOR plays an important role in cell growth and proliferation [93] and its activation was recently associated to intestinal inflammation induced by *C. jejuni* [94]. U937 cells were pre-treated with RM 100 nM for 2 h before the treatment with lysates.

### 4.4. Pre-Treatment of U937 Cells With C. jejuni Lysates

U937 cells were incubated for 6, 12, 24, 48, and 72 h with 2 mL of media enriched with *C. jejuni* cell lysates (1:100 dilution) from ATCC 33291 and 11168H *cdtA* mutant strains previously prepared. Treated cells were analyzed by means of flow cytometry and confocal microscopy to evaluate different cellular parameters. For the negative control, cells were incubated with media only.

### 4.5. Detection of Cytotoxin Activity in C. jejuni Lysates

U937 cells were seeded into 24-well tissue culture plates at a density of 2.6 × 10^5^ cells per well for 2 h prior to the addition of the lysates diluted at 1:5, 1:10, 1:50, 1:100, and 1:500, respectively. The cultures were incubated at 37 °C in 5% CO_2_ atmosphere and examined microscopically at 24-hour intervals for 4 days to evaluate morphological changes. The best acting dilution was the 1:100 which was chosen for all subsequent evaluations. All strains were tested in at least three independent experiments.

### 4.6. Morphological Feature Evaluation

To evaluate changes in cell morphology and size, both cytometry and confocal microscopy were used. In flow cytometry, populations that differed in size and morphology were distinguished by using their physical characteristics: forward scatter (FSC, cell size) and side scatter (SSC, cell granularity). Although a quantitative analysis was carried out by flow cytometry, a parallel microscopic evaluation of cell dimension was performed.

### 4.7. Flow Cytometry (FCM) and Confocal Microscopy (CM) Stainings

#### 4.7.1. Flow Cytometric Detection of Cell Death and Flow Cytometric Absolute Count

Cell death features were evaluated using supravital propidium iodide (PI) (Sigma-Aldrich) that is capable of binding and labeling DNA. No permeabilized cells were incubated 30 min in the dark with 50 μg/mL PI. Cells were washed with PBS and then analyzed by flow cytometry. Apoptotic and necrotic cells were detected as PI_dim_ and PI_bright_ clusters, respectively.

To investigate programmed cell death features (early and late apoptotic, as well as necrotic cells), we also used a double staining FITC-conjugated Annexin V-PI (AnxV-PI). AnxV allows to detect phosphatidylserines exposed on the outer cell membrane following caspase activation. AnxV-PI staining was carried out according to the manufacturer’s instructions (Immunostep, Spain). Absolute cell counting was performed by using Dako CytoCount^TM^ beads (Thermo Fisher Scientific, Waltham, MA, USA). 200 μL of sample was carefully dispensed at the bottom of the tube and 50-μL beads were added. Samples were acquired by using a FACSCanto II cytometer ((BD, Franklin Lakes, NJ, USA)) within 60 min. Approximately 20,000 cell events were collected. Setup and calibration procedures were optimized for the absolute counting protocols [95].

#### 4.7.2. Evaluation of Cellular Division by CFSE Staining

Carboxyfluoresceinsuccinimidyl ester (CFSE) is a fluorescent dye that can measure cell proliferation using flow cytometry. CFSE was transported into the cell during incubation with mononuclear cells binding covalently to cytoplasmic proteins, without adversely affecting cellular function. Analysis of cell division can be determined through measuring CFSE intensity by flow cytometry: with each cell division, the fluorescent intensity per cell division is reduced by 50 %, thus providing a readout of the mitotic activity within a specific population of cells. U937 cells were staining with 3 µM CFSE at day 0 and then analyzed at 24, 48, and 72 h [54]. At these times, U937 treated and untreated samples were harvested and acquired by flow cytometry.

#### 4.7.3. Assessment of Lysosomal Involvement

To label and trace lysosomes in U937 cells, the acidotropic dye LysoTracker Deep Red (LTDR) (Thermo Fisher Scientific, Waltham, MA, USA) was used. LysoTracker probes are fluorescent acidotropic probes for labeling and tracking acidic organelles in live cells: it means that the amount of fluorescence obtained from staining with LysoTracker is directly related to the volume of lysosome-related organelles in a cell [96]. Cells were cultured at 37 °C and resuspended in pre-warmed (37 °C) medium containing 100 nM of LysoTracker (diluted in RPMI). After 45 min of incubation, red lysosomal fluorescence was detected by flow cytometry and confocal microscopy [97].

#### 4.7.4. Determination of Mitochondria and Mitochondrial Membrane Potential (∆Ψm)

Mitochondrial features were investigated by Tetramethylrhodamine ethyl ester perchlorate (TMRE) (Sigma-Aldrich, St. Louis, MO, USA) is a ∆Ψm-specific stain able to selectively enter the mitochondria depending on ∆Ψm, producing red fluorescence. TMRE 40 nM was added to the samples 15 min before the acquisition time. The samples were analyzed by confocal microscopy and flow cytometry using the appropriate fluorescence channel [45,98].

#### 4.7.5. ER Stress Evaluation

ER-Tracker Green (Thermo Fisher Scientific, Waltham, MA, USA) is a live-cell stain highly selective for the ER. This stain consists of the green-fluorescent BODIPY^®^ FL dye and glibenclamide that bind to the sulphonylurea receptors of ATP-sensitive K^+^ channels which are prominent on the ER and have a critical role in the ER luminal homeostasis. Indeed, ER K^+^ channels are involved in functions such as protein folding, apoptosis, and Ca^2+^ homeostasis [99,100].

U937 were incubated with 100 nM ER-Tracker Green for 30 min at 37 °C and subjected to flow cytometry and confocal analyses.

#### 4.7.6. CD317 Expression Evaluation

To evaluate CD317 expression, fluorochrome-conjugated monoclonal antibodies were added to 50 μL of cell pellet. Mouse monoclonal anti-human antibodies Alexa Fluor^®^ 647 anti-CD317 (129C1) (BioLegend, San Diego, CA, USA) was added at dilutions according to the manufacturer’s instructions. After 15 min of incubation at RT, samples were acquired by flow cytometry and/or confocal microscopy.

#### 4.7.7. Intracellular Detection of Prb and Bcl-2 Antigens

U937 were washed in PBS for 10 min at RT, resuspended in 300 μL formaldehyde 3.7% and incubated at 4 °C for 15 min; 2 mL perm/washing buffer was added and cells were centrifuged at 1200 rpm for 10 min. Pellets were resuspended in 300 μL Cytoperm reagent [101]. Monoclonal anti-human antibodies Anti-pRb PE conjugated (clone G3-245) (BD) and anti bcl-2 FITC (clone 124) (Dako, Milan, Italy) were added to samples at concentrations according to the manufacturer’s instructions. Cells were incubated at 4 °C for 30 min before being processed by flow cytometry and/or confocal microscopy.

#### 4.7.8. Extracellular Vesicle Detection

In order to detect microparticles in the extracellular environment, without any preparation step of ultracentrifugation, we resuspended each sample (20 μL) in 0.22-filtered PBS 1X. We performed the test by using a BD custom product, i.e., a kit based on an APC emitting lipophilic cationic dye that diffuses through the plasma membranes [102]. After an incubation of 60 min to ensure the binding of the monoclonal antibody (CD107a mAb, clone H4A3) (BioLegend, London, UK) to the specific epitope, we proceeded with FC analyses. The flow cytometry approach consisted of acquiring samples mixed with beads of defined size (Ø 0.5 μm, 1 μm, 5.2 μm) to obtain a size calibration of small particles detected outside the scatter area of intact cells. Furthermore, it is important to specify that the FCS of the complete medium was ultracentrifuged to minimize contamination by serum microvesicles. Samples were acquired by a FACSCanto flow cytometer and a FACSDiva™ software was used to analyze all data.

#### 4.7.9. Cytometric Investigations

Cytometric experiments were carried out with a FACSCanto II flow cytometer (BD, Franklin, Lakes, NJ, USA) equipped with an argon laser (Blue, Excitation 488 nm), a helium-neon laser (Red, Excitation 633 nm), and a solid state diode laser (Violet, Ex 405 nm). Analyses were performed with the FACSDiva^TM^ software (BD); approximately 10,000 cell events were acquired for each sample.

#### 4.7.10. Confocal Microscopy Analyses

Confocal microscopy analyses were applied by a Leica TCS SP5 II confocal microscope (Leica Microsystem, Germany) with 488, 543, and 633 nm illuminations and oil-immersed objectives. For confocal live imaging, cells were grown on MatTek glass bottom chambers (MatTek Corporation, Bratislava, Slovak Republic). The images were further processed and analyzed in ImageJ software (NIH, Bethesda, MD, USA).

### 4.8. RNA Isolation and cDNA Synthesis

Cells were directly lysed with 700 μL of QIAzol^®^ Lysis Reagent (Qiagen, Hilden, Germany). Total RNA extraction was performed with the miRNeasy Mini Kit (Qiagen, Hilden, Germany) following the manufacturer’s instructions. The extracted RNA was quantified using a NanoVue Plus^TM^ spectrophotometer (GE Healthcare Life Sciences, Piscataway, NJ, USA). The cDNA synthesis was performed from 500 ng of total RNA using PrimeScript^TM^ RT Master Mix (Perfect Real Time) (Takara Bio Inc. Shiga, Japan) according to the manufacturer’s instructions.

### 4.9. Quantitative Real-Time PCR

The expression of CHOP was monitored by qPCR using RT² SYBR Green ROX FAST Mastermix (Qiagen, Hilden, Germany) in a RotorGene 6000 instrument (Corbett life science, Sydney, Australia), as described previously [103]. A non-template control was included in duplicate for each primer pair reaction, as a negative control. To exclude the presence of non-specific products or primer dimers, a melting curve analysis from 65 °C to 95 °C was performed at the end of each run. GUSB (Beta-D-Glucuronidase) gene was used as reference. Relative mRNA expression was calculated using the comparative quantitation analysis of the RotorGene 6000 software.

### 4.10. Statistical Analyses

Data are shown as mean (or percentage, as indicated) ± standard deviation (SD) of at least three independent experiments. Analyses of variance (ANOVA) approaches were used to compare values among more than two different experimental groups for data that met the normality assumption. One-way ANOVA or two-way ANOVA were followed by a Bonferroni post-hoc test. The means of two groups were compared by using a t test. The *p* values less than 0.05 were considered statistically significant. All statistical analyses were performed using GraphPad Prism 5.0 (GraphPad software, San Diego, CA, USA).

## Supplementary Materials

The following are available online at https://www.mdpi.com/1422-0067/21/6/2207/s1.

## Abbreviations

*C. jejuni*	*Campylobacter jejuni*
AnxV	Annexin V
Atg	Autophagy-Related Proteins
CDT	Cytolethal Distending Toxin
CFSE	Carboxyfluoresceinsuccinimidyl ester
ER	Endoplasmic Reticulum
EVs	Extracellular vesicles
GBS	Guillain-Barré syndrome
LTDR	LysoTracker Deep Red
MFI	Mean Fluorescence Intensity
MVB	Multivesicular body
pRb	Retinoblastoma Protein
ReA	reactive arthritis
RM	Rapamycin
TGN	Trans-Golgi Network
TMRE	Tetramethylrhodamine ethyl ester perchlorate
UPR	Unfolded protein response
