# Supplementary material for: Rapamycin Re-Directs Lysosome Network, Stimulates ER-Remodeling, Involving Membrane CD317 and Affecting Exocytosis, in Campylobacter Jejuni-Lysate-Infected U937 Cells
Source: Int J Mol Sci. 2020 Mar 23;21(6):2207. doi: 10.3390/ijms21062207 (PMC7139683; doi:10.3390/ijms21062207)
Supplement: Supplementary file 1 [file ijms-21-02207-s001.pdf]

SUPPLEMENTARY MATERIAL

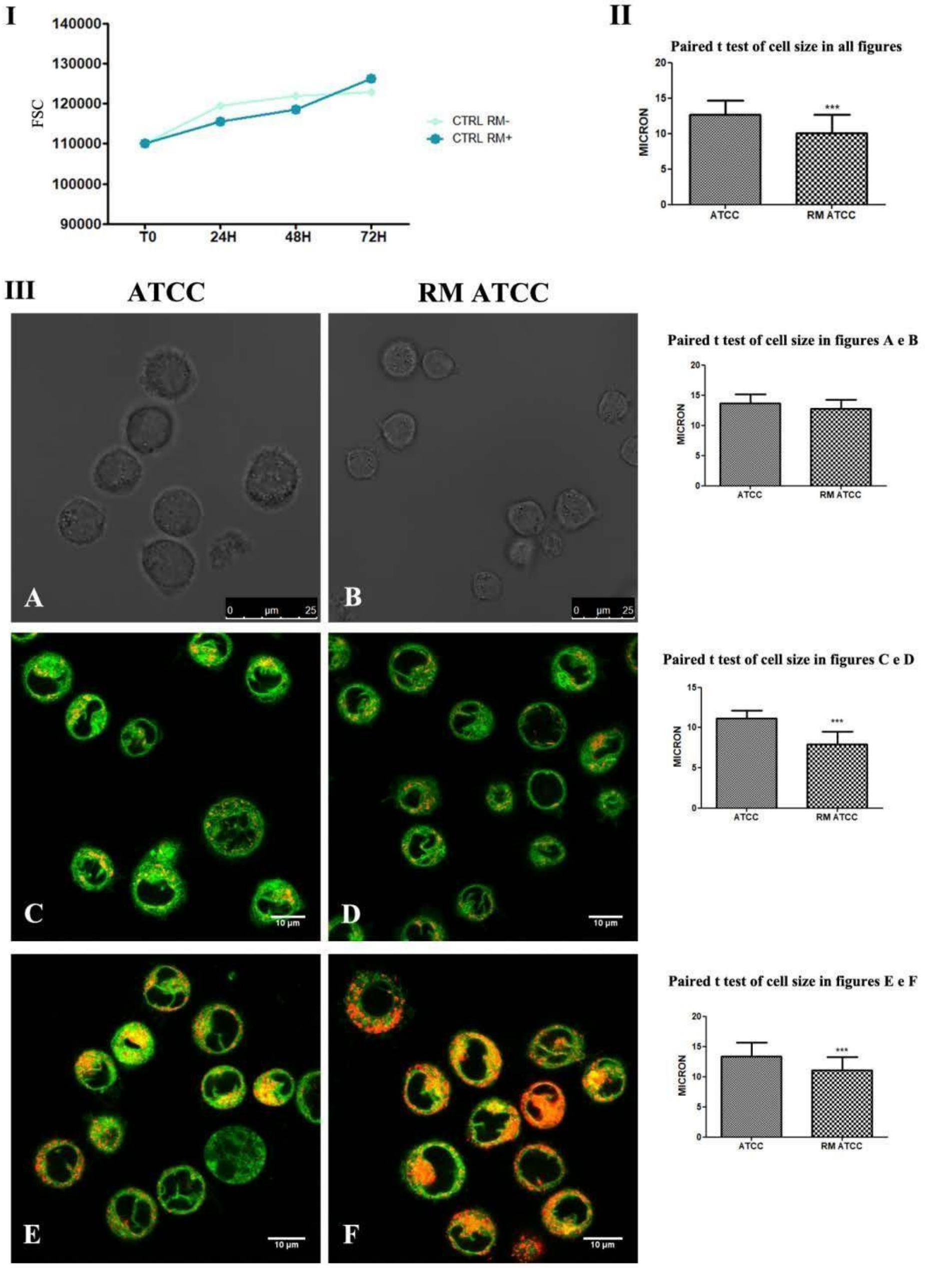

Figure 1. Evaluation of cell size induced by lysates and RM administration **I** Trends of forward light scatter (FSC) values for control cells with (RM+) or without (RM-) Rapamycin, during the time course from the starting point (T0) to 72h. Each value is expressed as a mean ± SD (Results from $n \geq 3$ independent experiments). **II** Statistical histograms on cell size evaluated by microscopic measurements, comparing ATCC33291 lysate treated U937 cells (ATCC) and the same cells pre treated with Rapamycin (RM ATCC). Paired t test (two-tailed *p* value) revealed: *** $p < 0.0001$ ATCC 33291vs RM ATCC 33291. **III** Single confocal optical sections of ATCC33291 lysate treated U937 cells (ATCC) and the same cells pre treated with Rapamycin (RM ATCC), from different analyses: **A-B**: Brightfield, **C-D**: ER Tracker/TMRE; **E-F**: ER tracker/LTDR utilized for cell size measurements.
